# Cognitive impairment and associated white matter organization abnormalities across the illness course of schizophrenia

**DOI:** 10.1007/s00406-025-02161-2

**Published:** 2025-12-08

**Authors:** Chen-Lan Shen, Shih-Jen Tsai, Ching-Po Lin, Albert C. Yang

**Affiliations:** 1 Institute of Brain Science, National Yang Ming Chiao Tung University, No.155, Sec. 2, Linong St. Beitou Dist, Taipei, 112304 Taiwan; 2 Department of General Psychiatry, Tsao-Tun Psychiatric Center, Nan-To, Taiwan; 3 School of Medicine, National Yang Ming Chiao Tung University, Taipei, Taiwan; 4 Department of Psychiatry, Taipei Veterans General Hospital, Taipei, Taiwan; 5 Institute of Neural Science, National Yang Ming Chiao Tung University, Taipei, Taiwan; 6 Department of Medical Research, Taipei Veterans General Hospital, Taipei, Taiwan

**Keywords:** Schizophrenia, Cognitive deterioration, White matter disintegration, Illness course

## Abstract

**Aim:**

Schizophrenia is a chronic brain disorder with cognitive impairment as one of the cardinal manifestations. White matter organization abnormalities shown on brain imaging were also documented in patients with schizophrenia. However, when and how the cognitive impairment, the white matter organization abnormalities and their correlation occur and evolve throughout the illness course of schizophrenia remain undetermined. We aimed to show cognitive impairment and white matter organization abnormalities across different stages of schizophrenia and the association between them.

**Methods:**

We collected 4 groups of participants: 40 individuals with illness duration of schizophrenia below 5 years, 36 individuals with illness duration of schizophrenia around 15 years, 39 individuals with illness duration of schizophrenia above 25 years, and 78 healthy controls. All participants underwent 3 cognitive tests (Wisconsin Card Sorting Test, Digit Span, Mini-Mental Status Examination) and 2 diffusion tensor imaging analyses of white matter (fractional anisotropy and graph theoretical analysis).

**Results:**

We found cognitive impairment aggravated from early stage to the third decade of illness in schizophrenia. The associated white matter structural abnormalities had two steps of change: alteration in network organization occurred at early stage of schizophrenia, and then reduction in white matter tract integrity ensued from the second decade of the illness. The white matter integrity reduction was associated with cognitive impairment.

**Conclusion:**

By using groups of participants with different illness duration, we found cognitive impairment and white matter organization abnormalities and their association across a three-decade illness course in schizophrenia. Our findings suggested active cognitive rehabilitation to patients with schizophrenia throughout the course of illness may be warranted.

**Supplementary Information:**

The online version contains supplementary material available at https://doi.org/10.1007/s00406-025-02161-2.

## Introduction

As its original name dementia praecox indicates, cognitive deterioration is one of the hallmark manifestations of schizophrenia [1, 2]. Many patients with schizophrenia are impaired in self-care ability and social or occupational functioning due to cognitive difficulties despite of long-term antipsychotics treatment and rehabilitation [3].

Among cognitive domains, executive function is involved in information selecting, inhibiting and shifting and was shown to be impaired in patients with schizophrenia on the Wisconsin Card Sorting Test (WCST) [4–6]. Impairment in the WCST performance is present before antipsychotics treatment, in acute stage and in chronic stage of schizophrenia, [7, 8] but whether executive function deteriorates continuously, halts at some points or improved gradually throughout the illness course remained controversial [9].

Working memory is the cognitive ability of holding information temporarily for further manipulating and can be assessed with the forward digit span (DSF) task or the backward digit span (DSB) task [10]. The DSF task and the DSB task were reported be impaired in patients with schizophrenia, and DSB was also abnormal in their first-degree relatives, implicating its genetic and biological significance in pathogenesis [11]. Apart from specific cognitive domains, general cognitive ability reflected by the Mini-Mental Status Examination (MMSE) has been known to deteriorate in patients with schizophrenia living in communities and long-term facilities [12]. However, whether working memory impairment and general cognitive ability deficit progress until decades later in patients with schizophrenia are rarely studied since most long-term studies focused on psychopathological outcomes rather than cognitive deficits [13].

One of promising theories about pathogenesis of schizophrenia is the disconnection hypothesis, which attributes the illness cause to wrong wiring of the brain [14]. Since brain regions are wired with nerve fibers, impairment in integrity and connectivity of white matter tracts may be associated with clinical manifestations of schizophrenia, including cognitive impairment [15–17]. By using DTI, integrity of individual white matter tracts can be measured with fractional anisotropy (FA) and connectivity of white matter tracts can be quantified with graph theoretical analysis in patients with schizophrenia [18–20]. Reductions in FA of almost all white matter tracts has been found in patients with schizophrenia at early and chronic stages [21, 22]. However, how the FA reductions evolve throughout the illness course remains debatable, with several studies revealing white matter integrity deficits in limited brain areas at early stage of schizophrenia and widespread changes at chronic stage [23, 24]. Previous studies also reported correlation of FA impairment and the WCST in patients with schizophrenia but the findings were inconsistent [25, 26]. Deficits in working memory and general cognitive ability were shown to correlate with FA levels, but the DSF task, the DSB task, and the MMSE were rarely used as cognitive tests in those studies [17, 27, 28]. In contrast to white matter tracts integrity, white matter network organization can be studied by using graph theoretical analysis [29–31]. Conceptually, the former means ‘corrosion of wires’ and the latter reflects ‘disorganization of wiring’. Several graph theoretical metrics were shown to alter in patients with schizophrenia but the findings are divergent [32, 33]. Whether alterations in network organization occur earlier or later than reductions in white matter integrity and whether they aggravate at later illness stages have never been studied.

Accordingly, the present study aimed to explore cognitive impairment, white matter organization abnormalities, and their relationship across the illness course of schizophrenia. We used three cognitive tests (WCST, DSF & DSB, MMSE) and two DTI analysis methods (FA, graph theory) to investigate three groups of adults with different illness durations of schizophrenia. Since longitudinal follow-up for three decades is difficult, our cross-sectional study is a feasible alternative to delineate long-term cognitive outcomes and their white matter correlates in schizophrenia.

## Methods

### Participants

All the participants were collected from the Taiwan Aging and Mental Illness Cohort (TAMI) dataset [34]. The individuals with schizophrenia were recruited from the Department of Psychiatry at Taipei Veterans General Hospital, Taiwan. Two board‐certified psychiatrists diagnosed all the individuals with schizophrenia using the diagnostic criteria of the *Diagnostic and Statistical Manual of Mental Disorders, Fourth Edition Text Revision* (*DSM-IV-TR*). The Mini‐International Neuropsychiatric Interview (MINI) [35] was used to validate diagnoses of schizophrenia, and the Positive and Negative Syndrome Scale (PANSS) to evaluate psychotic symptoms [36]. Chlorpromazine (CPZ) equivalent dosages were calculated according to the American Psychiatric Association guidelines [37].

The healthy controls were recruited from a healthy cohort from local communities. To exclude the presence of psychiatric disorders, the healthy controls were evaluated by a trained research assistant using the MINI. The healthy controls had no personal or family history (first‐degree relatives) of psychiatric disorders. The following were the exclusion criteria for both groups: head trauma or neurological disease history, severe medical illness, or substance abuse history. This study was conducted in accordance with the Declaration of Helsinki and was approved by the Institutional Review Board of Taipei Veterans General Hospital and National Yang Ming Chiao Tung University. Informed consent was obtained from all the participants. The data derived from the TAMI has been quality-controlled, and subjects with poor imaging quality have already been excluded from the database.

### Image acquisition and processing

DTI scanning was performed at National Yang Ming Chiao Tung University by using a 3.0T Siemens MRI scanner (Siemens, Erlangen, Germany) equipped with a 12‐channel head coil. We acquired whole-brain DTI images by using a single-shot spin-echo EPI sequence in the axial-plane and the parameters were: repetition time = 11,000 ms; echo time = 104 ms; matrix size = 128 × 128; number of excitations = 3; field of view = 26 cm; slice thickness = 2.0 mm; slice number = 70; b-value = 1000 s/mm^2^; isotropic diffusion directions = 30; non-diffusion weighted T2 images = 3.

We used the Functional Magnetic Resonance Imaging of the Brain (FMRIB) diffusion toolbox, included in the Functional Magnetic Resonance Imaging of the Brain Software Library (FSL) to preprocess raw DTI images [38]. The effects of head movement and eddy currents were then corrected and non-brain components of each image were removed with a brain extraction tool [39]. We calculated FA values by fitting a tensor model to the data at each voxel from these images. The Tract-Based Spatial Statistics (TBSS, part of FSL) pipeline was used to register and normalize the FA images to the MNI standard space used to target the FMRIB58 FA standard-space image [40]. We performed a total of 5000 permutations for each group comparison. A t-test was used to conduct group comparisons. The p-value images were fully corrected by threshold-free cluster enhancement (TFCE) and p was set at < 0.05 [41]. We developed a mean FA skeleton to represent the centers of all tracts common to the group by creating and thinning a mean FA image of all the participants. To exclude non-skeletal voxels, an FA threshold of 0.2 was applied. The FA data were aligned and then projected onto this skeleton for all participants, and the results underwent further statistical analyses. Voxel-wise analyses were performed using the TBSS.

### Connectivity matrix and network indices

We used the software DSI studio to produce connectivity matrix and conduct network analysis [42]. The diffusion image data were reconstructed with the generalized q-sampling imaging method and the diffusion sampling length ratio was 1.25. The tensor metrics were calculated using diffusion weighted imaging with b-value lower than 1750s/mm2. To improve reproducibility a, deterministic fiber tracking algorithm was used and the anisotropy threshold was randomly selected. The angular threshold was randomly selected within the range of 15 degrees to 90 degrees. The step size was randomly selected within the range of 0.5 voxel to 1.5 voxels. Tracks which were shorter than 30 mm or longer than 200 mm were discarded. A total of 1,000,000 seeds were placed.

Automated Anatomical Labeling (AAL) atlas [43] was used as the brain parcellation template, and connectivity matrix was calculated by counting the connecting tracks. A total of 116 nodes, corresponding to all the AAL brain areas, were used. To binarize the edges, connectivity matrix was thresholded by a track count in proportional to the max count 100,000 and the threshold was set at 0.01, which means an entry with 1,000 track count or more was set to 1, and any lower value was set to 0. There were 6,728 possible connections between any two brain areas, but only 1,626 remained after connectivity matrix was computed. Graph theoretical analysis was performed and four network indices were calculated: clustering coefficient, local efficiency, characteristic path length, and global efficiency [44]. These indices were reported to be associated with cognitive function in schizophrenia and other mental illnesses [45, 46]. The clustering coefficient is the fraction of triangles around a node and is equivalent to the fraction of node’s neighbors that are neighbors of each other. The local efficiency is the global efficiency computed on the neighborhood of the node, and is related to the clustering coefficient. The characteristic path length is the average shortest path length in the network. The global efficiency is the average inverse shortest path length in the network [47]. Previous studies showed local and global efficiency represents a network’s ability to support parallel processing, fault tolerance, and local and large scale functional integration. Local efficiency is associated with clustering coefficient, and global efficiency is related to path length [48].

### Cognitive tests

The Wisconsin Card Sorting Test, the Forward Digit Span task, the Backward Digit Span task, and the Mini Mental Status Examination were administered to all participants by a clinical psychologist on the same day as image scanning.

### Statistical analysis

We used IBM SPSS v22 as statistical software.

#### Demographic and clinical data

For calculating group differences in demographic and clinical data, a chi-square test or a t-test was performed as appropriate.

#### Cognitive tests and DTI images

For calculating group differences in cognitive test results, GLM-ANCOVA was used, with age, gender, education level, illness onset age, and cumulative exposure to antipsychotics as covariates. Post-hoc Turkey’s test was used for multiple comparison. GLM-ANOVA was used to calculate linear trends of cognitive test results of the three schizophrenia groups. We adjusted the p value by using FDR correction with its significance level was set at 0.05 [49].

In line with DTI image processing, since we were concerned with differences between each schizophrenia group and the control group, we used a t-test to compare DTI FA data. Voxel-wise analyses were performed using the FSL TBSS, and then we examined DTI FA values and regional differences in whole-brain parametric mapping between the schizophrenia groups and the healthy controls by extracting regional averages of FA values. We then chose the anatomical regions of interest according to 48 white matter track labels defined in the ICBM-DTI-81 atlas developed at Johns Hopkins University [50]. We calculated the average FA of each white matter tract by overlaying the group-specific white matter skeleton on the ICBM-DTI-81 atlas with a specific white matter label. We used a t-test to assess between-group differences in FA and used p < 0.001 to assess between-group difference in FA measures (i.e., 0.05/48 tests by applying the Bonferroni correction). All FA of the 48 white matter tracts were averaged to obtain mean FA and represented global white matter integrity [51].

For calculating DTI image graph indices, we also used a t-test.

#### Association between cognitive tests and DTI image data

For calculating association between cognitive test results and DTI image data (FA and graph indices), GLM-regression and Pearson’s correlation analysis was performed.

The three schizophrenia groups were combined into one group and Pearson’s correlation analysis was conducted between mean FA of individual white matter tracts FA and cognitive test results.

As to the GLM-regression, the model was: cognitive test result 〜 mean FA + group (or + illness duration) + mean FA × group (or + mean FA × illness duration) + age + education level + gender + illness onset age + cumulative exposure to antipsychotics. The mean FA is the average FA of the the 48 white matter tracts. Cognitive test results were dependent variables, mean FA and group (or illness duration) were independent variables, and age, education level, gender, illness onset age and cumulative exposure to antipsychotics were covariates. We set the significance threshold at 0.05, two-tailed.

## Results

### Participants

This study incorporated 193 Han Chinese participants and included four groups: 5-year SZ, 15-year SZ, 25-year SZ and HC. The 5-year SZ consisted of 40 individuals with schizophrenia for less than 5 years (20 men and 20 women; mean age: 36 ± 11.8 y); the 15-year SZ consisted of 36 individuals with schizophrenia for around 15 years (21 men and 15 women; mean age: 44.4 ± 6.4 years); the 25-year SZ consisted of 39 individuals with adults for around 25 years (23 men and 16 women; mean age: 54.1 ± 8.5 y); and the HC had 78 healthy controls (43 men and 35 women; mean age: 43.2 ± 7.9 y). The participants’ demographic and clinical data are listed in Table 1. Age at scan, sex ratio and education level were different among the four groups of participants. We observed no significant difference in PANSS scores or CPZ equivalence between the individuals with schizophrenia in the 5-year SZ, 15-year SZ and 25-year SZ groups. 17% of all the recruited individuals with schizophrenia were being hospitalized in a psychiatric ward and 83% were outpatients. 93% of the individuals with schizophrenia were in remission [52].

**Table 1:** Demographic and Clinical Characteristics

	HC (n = 78) Mean (SD)	5-year SZ (n = 40)	15-year SZ (n = 36)	25-year SZ (n = 39)	p
		Mean (SD)	Mean (SD)	Mean (SD)	
Age at scan (years)	43.2(7.9)	36(11.8)	44.4(6.4)	54.1(8.5)	0.02
Sex, female	43(56%)	20(50%)	21(63%)	23(58%)	0.03
Handedness, right	75(97%)	35(97%)	30(97%)	37(96%)	0.93
Education	15.7(5.2)	12.9(6.9)	13.2(5.4)	11.6(6.1)	0.02
Age of onset		33(11.8)	28.7(7.9)	25(7.3)	0.03
Illness duration		2.54(0.4)	15.7(1.4)	29.7(7.5)	0.01
PANSS total		38.3(9.2)	37.3(9.2)	39.3(9.2)	0.82
PANSS positive		9.7(3.2)	8.6(3.3)	9.2(3.5)	0.86
PANSS negative		9.3(5.2)	9.5(5.7)	9.7(5.1)	0.92
PANSS general		22.3(3.9)	21.3(3.7)	22.5(3.8)	0.89
CPZ equivalence		495(390)	425(312)	398(278)	0.87

*SZ* individuals with schizophrenia, *HC* healthy controls, *PANSS* Positive and Negative Syndrome Scale, *CPZ* chlorpromazine

p < 0.05

### Cognitive tests

**Fig. 1:**
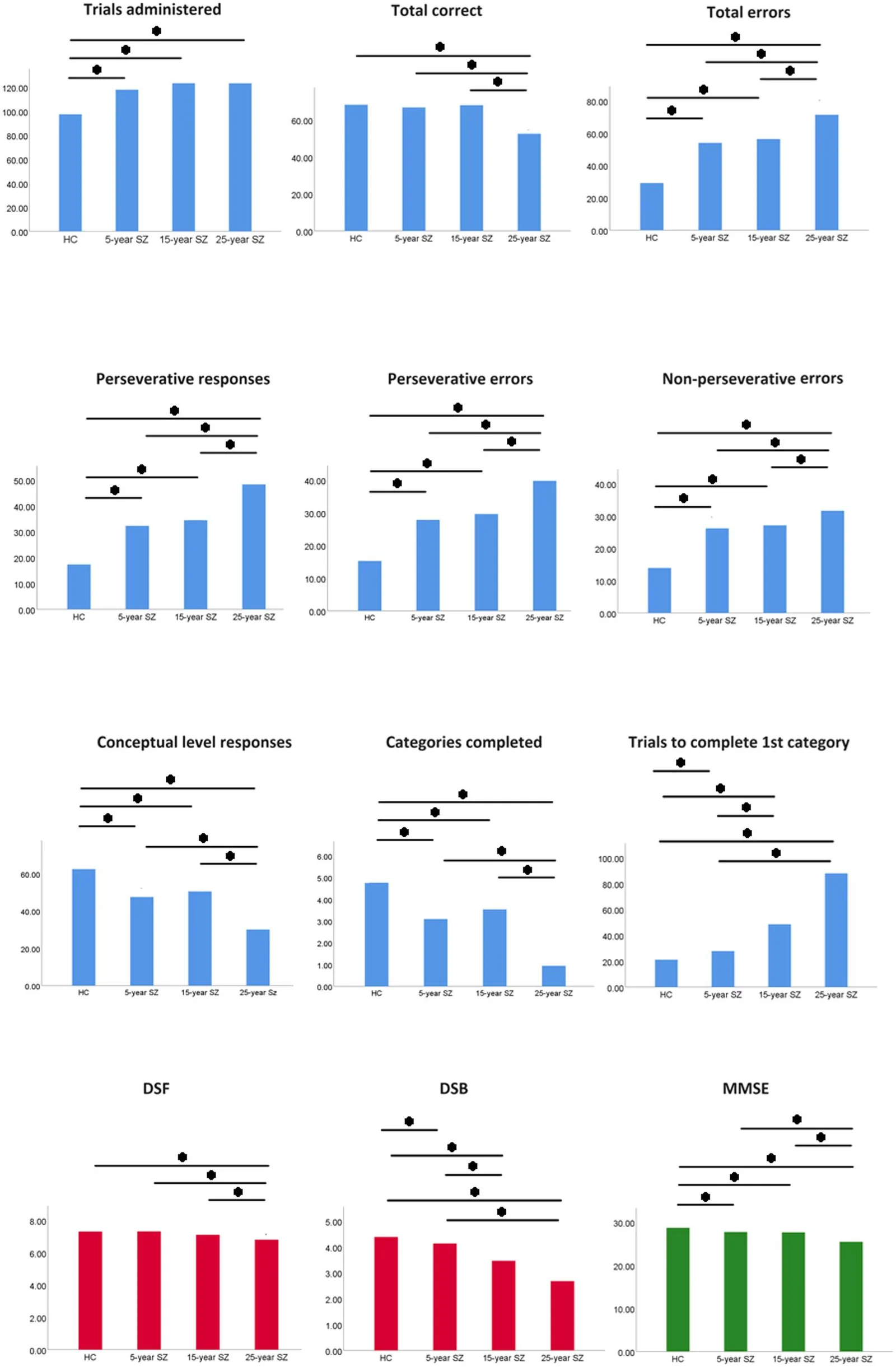
Results of cognitive tests. *MMSE* Mini Mental Status Examination, *DSF* digital span forward, *DSB* digital span backward, *SZ* individuals with schizophrenia, *HC* healthy controls. Black horizontal lines indicate significant between-group difference. The asterisks represent adjusted p (corrected with FDR) < 0.05

The cognitive test results are presented in Fig. 1 and Table 2. Of the cognitive tests, 9 items of the WCST, DSB and MMSE showed significant between-group difference, and only DSF showed insignificant result. As for linear trends, Total correct, DSF and MMSE showed negative findings and all other items of the cognitive tests were statistically significant. Items with significant post-hoc between-group differences were highlighted with black horizontal lines and asterisks on Fig. 1

.

**Table 2:** Results of cognitive tests

	HC	5-year SZ	15-year SZ	25-year SZ	Adjusted p (between groups)	Adjusted p (linear trend)
*WCST*						
Trials Administered	97.6 (30.6)	116.9 (10.1)	123.6 (4.6)	122.2 (5.7)	0.03	0.001
Total correct	68.2 (23.1)	63.8 (20.4)	66.8 (21.3)	53.9 (16.7)	0.04	0.334
Total errors	29.5 (9.3)	54.0 (17.6)	56.54 (18.9)	68.2 (22.1)	0.02	0.002
Perseverative responses	17.4 (5.4)	32.3 (10.2)	34.4 (11.4)	45.6 (14.6)	0.03	0.03
Perseverative errors	15.3 (4.6)	27.8 (8.3)	29.6 (9.3)	37.8 (12.3)	0.01	0.001
Non-perseverative errors	14.1 (3.5)	26.1 (7.5)	27.1 (8.5)	30.4 (9.6)	0.02	0.002
Conceptual level responses	60.2 (19.2)	47.4 (15.3)	50.4 (16.7)	32.5 (11.4)	0.02	0.03
Categories completed	5.0 (0.6)	3.0 (3.0)	3.5 (2.5)	1.2 (1.2)	0.03	0.02
Trials to completed 1st category	21.5 (6.8)	40.0 (13.5)	48.4 (15.7)	79.7 (23.4)	0.01	0.02
*Digit span*						
DSF	7.3 (2.4)	7.2 (2.4)	7.0 (2.1)	6.8 (1.9)	0.13	0.74
DSB	4.5 (1.6)	4.0 (1.5)	3.3 (1.1)	2.8 (0.8)	0.02	0.03
*MMSE*						
	28.7 (1.3)	27.4 (2.7)	27.1 (2.9)	25.7 (3.8)	0.03	0.51

*WCST* Wisconsin Card Sorting Test, *DSF* Forward Digit Span, *DSB* Backward Digit Span, *SZ* individuals with schizophrenia, *HC* healthy controls. Adjusted p (corrected with FDR) < 0.05

### FA analysis and correlation of FA with cognitive function

**Fig. 2:**
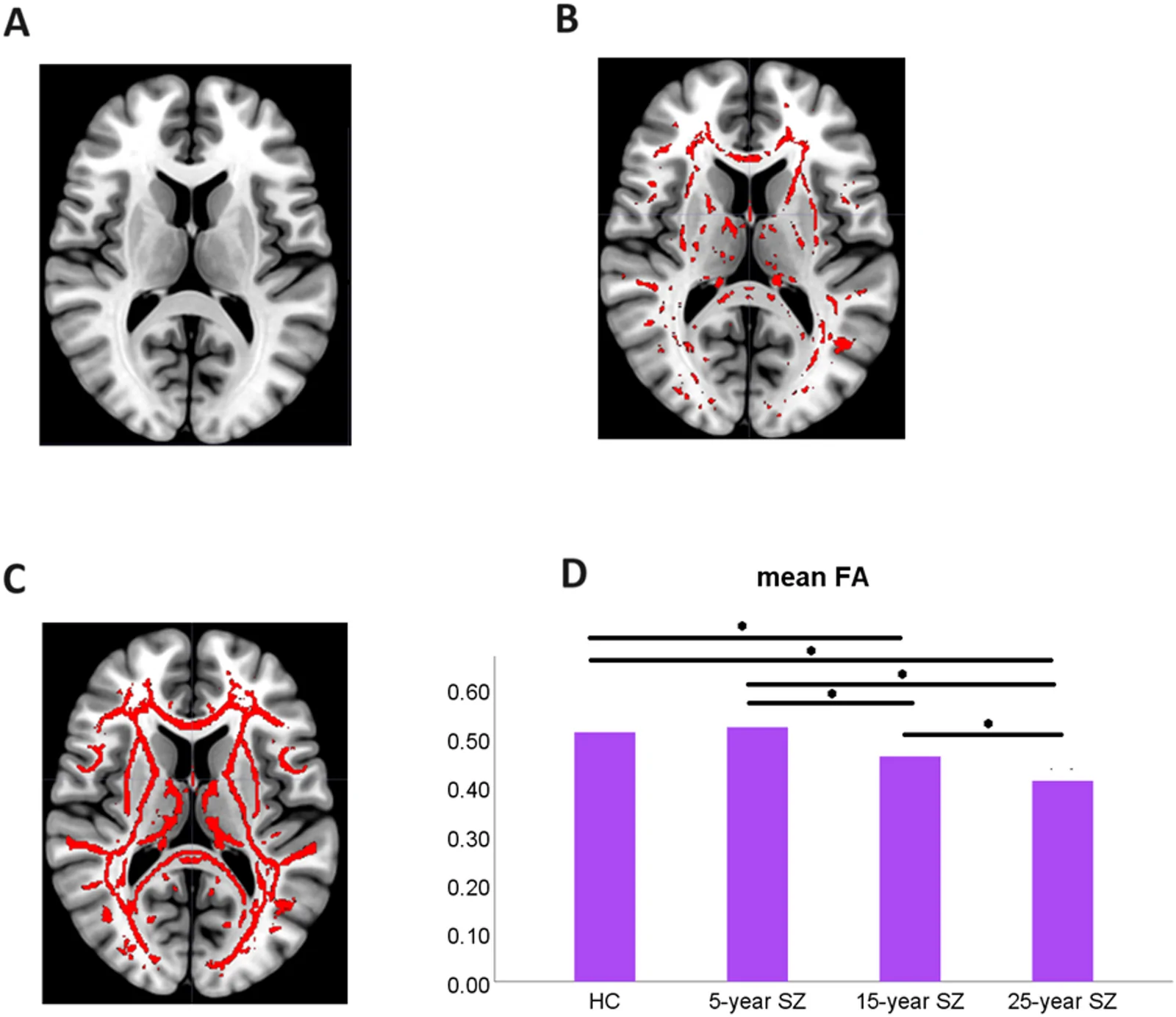
White matter tracts of the individuals with schizophrenia with FA significantly different from the healthy controls and their mean FA. White matter tracts with significantly different FA are depicted in red lines. A: 5-year SZ compared with HC. B: 15-year SZ compared with HC. C: 25-year SZ compared with HC. D: mean FA of the four groups. Black horizontal lines indicate significant between-group difference. The asterisks represent *p* < 0.05. *SZ* individuals with schizophrenia, *HC* healthy controls

As shown in Fig. 2A, B and C, compared with HC, 5-year SZ showed no difference in FA of any white matter tract, 15-year SZ showed differences in FA of several white matter tracts (genu of corpus callosum, body of corpus callosum, splenium of corpus callosum, left anterior corona radiata, left superior corona radiata, right cingulum cingulate gyrus, left posterior limb of internal capsule) and 25-year SZ showed differences in FA of almost all white matter tracts. Complete FA data was listed in Supplement Table 1. As shown in Fig. 2D, we found mean FA decreased in 15-year SZ and further decreased in 25-year SZ, compared with HC.

White Matter Tracts with Significant Pearson’s Correlation of FA with WCST are listed in Supplement Table 2. Trial administered, Total correct, Perseverative responses, Perseverative errors, Non-perseverative errors, Conceptual level responses, Categories completed, Trials to complete 1st category and DSF were respectively associated with FA of several white matter tracts in the individuals with schizophrenia. Trials administered, Total correct, Total errors, Perseverative responses, Conceptual level responses, Categories completed, Trials to complete 1st category, DSB, and MMSE were also respectively associated with FA of several white matter tracts in the healthy controls, which are mostly different from the schizophrenia groups. In contrast, mean FA was associated with Total correct and Conceptual level responses both in the combined schizophrenia group and in the control group.

In Table 3 (Full results were listed on Supplement Fig. 1), GLM-regression analysis showed the adjusted model significantly predicted Total correct, Conceptual level responses and Trials to complete 1st category in the individuals with schizophrenia. The interaction item mean FA × group was significantly associated with Total correct, Conceptual level responses and Trials to complete 1st category. In the healthy controls, the adjusted model significantly predicted Total correct, Perseverative responses, and Conceptual level responses. However, when we replaced group with illness duration in the regression models, no significant results were obtained.

**Table 3:** Summary of general linear models which significantly predicted cognitive test results from mean FA

SZ			HC		
	F	p		F	p
Total correct			Total correct		
Adjusted model	2.1	0.035	Adjusted model	4.35	0.004
mean FA × group	4.7	0.004	R^2^	0.492	
R^2^	0.195				
Conceptual level responses			Conceptual level responses		
Adjusted model	2.04	0.04	Adjusted model	4.35	0.004
mean FA × group	3.91	0.01	R^2^	0.362	
R^2^	0.17				
Trials to complete 1st category			Perseverative responses		
Adjusted model	3.31	0.002	Adjusted model	3.29	0.018
mean FA × group	9.43	0.01	R^2^	0.362	

*SZ* individuals with schizophrenia, *HC* healthy controls

p < 0.05

### Network analysis

The results are presented in Table 4. Of the global network indices, clustering coefficient, local efficiency, and global efficiency were lower in 5-year SZ, 15-year SZ, 25-year SZ than in HC, but there were no differences between 5-year SZ, 15-year SZ or 25-year SZ; characteristic path length were higher in 5-year SZ, 15-year SZ, and 25-year SZ, but there were no differences between 5-year SZ, 15-year SZ or 25-year SZ.

**Table 4:** Global network indices calculated from FA of the healthy controls and the individuals with schizophrenia

	HC (SD)	5-year SZ(SD)	15-year SZ(SD)	25-year SZ(SD)	p
Clustering coefficient	0.76(0.03)	0.67(0.02)	0.64(0.03)	0.66(0.04)	0.04
Local efficiency	0.92(0.04)	0.84(0.03)	0.81(0.04)	0.82(0.03)	0.03
Characteristic path length	1.46(0.06)	1.72(0.07)	1.69(0.05)	1.74(0.06)	0.03
Global efficiency	0.84(0.05)	0.72(0.04)	0.69(0.05)	0.68(0.04)	0.02

*SZ* individuals with schizophrenia, *HC* healthy controls

p < 0 .05

### Effects of illness onset age and cumulative exposure to antipsychotics

Combining all the schizophrenia participants into one group, we analyzed the correlation of illness onset age with cognitive test results and FA values. We obtained no significant correlation in any white matter tract. In addition, by using dose-years (CPZ equivalent × years of taking antipsychotics) as the total amount of antipsychotics use, we found no white matter tract with a significant correlation between accumulative exposure to antipsychotics and white matter tract abnormalities in the schizophrenia groups.

## Discussion

### Cognitive impairment and white matter organization abnormalities across the illness course

To our knowledge, this is the first study to determine the chronological pattern of cognitive impairment and associated white matter organization abnormalities across the illness course of schizophrenia up to three decades. We used 3 groups of individuals with different duration of schizophrenia to represent the illness course and 3 cognitive tests and 2 white matter analysis methods to delineate the trajectories of cognitive and white matter changes. We chose a discreet grouping approach rather than a dimensional one because we examined the associations between illness duration and cognitive scores with age being controlled and obtained negative results. We found cognitive impairment aggravated from early stages to the third decade of illness and white matter organization abnormalities had two steps of change. Alteration in white matter network organization occurred at early stage of schizophrenia, and then reduction in white matter tract integrity ensued from the second decade of the illness on. The reductions in white matter integrity, manifesting both on individual and global levels, were associated with cognitive impairment in the adults with schizophrenia and the healthy controls.

Multiple cognitive domains have been shown to be impaired in schizophrenia and among the most studied are executive function, working memory and processing speed. We did not include processing speed because it is particularly affected by antipsychotic medications and deficits in this domain may reflect impairments in other higher-order functions [53]. WCST is the most widely used test for executive function, and executive function is crucial to social and occupational function of patients with schizophrenia [54, 55]. Previous studies extensively documented impaired WCST performance in schizophrenia but were inconclusive on its change along the illness course [56]. How cognitive impairment evolves after the onset of schizophrenia is still a matter of debate [57, 58]. This study, by showing WCST performance aggravated throughout a three-decade course, favored the neurodegenerative hypothesis of schizophrenia, [57, 58] and implied that cognitive remediation is beneficial even to patients having schizophrenia for decades [59]. Working memory deficits in schizophrenia were shown to have no correlation with duration of illness in previous studies, [60] but we found DSF and DSB performance declined further at later stages of schizophrenia. Previous studies attributed MMSE score reduction to accelerated aging in schizophrenia, but this study showed that illness duration still played a role after controlling the age factor [61, 62]. The participants with schizophrenia scoring higher in MMSE than previous studies may be due to lower PANSS scores (milder symptomatology) in this study [63]. We controlled the education level to exclude its influence on cognitive test performance [64].

Though white matter tract abnormalities in schizophrenia were documented since DTI analysis was developed three decades ago, their deterioration trajectory remains debatable [19]. Early studies showed impaired white matter integrity in first-episode schizophrenia, but recent studies found significant reductions in FA emerged from about age 35 and continued to deteriorate into the more chronic stages of schizophrenia [24, 65]. This study corroborated the latter findings and added that white matter tract abnormalities arose during the second decade of illness and had involved all most all tracts by the third decade. As to association of FA with cognitive function in schizophrenia, previous study results were equivocal, with some showing significant association in either schizophrenia groups or healthy people, or in both, [28, 66] and our study supported the latter findings. Previous studies on healthy adults or cognitively impaired elderly people have used mean FA to represent global white matter integrity, [51, 67] and our study suggested its integrity may have better association with cognitive function than individual white matter tracts in patients with schizophrenia and healthy people. Though mean FA was significantly associated with several WCST items, the R^2^ was below 0.5, which means it could only explained a small part of cognitive impairment in patients with schizophrenia. Gray matter abnormalities or deficit in integration of gray matter function and white matter organization may be other parts of the neural correlate to cognitive deterioration in schizophrenia. The interaction item mean FA × group was significantly associated with the WCST results in our study, which suggested illness duration might play additional roles in cognitive impairment beyond contributing to FA changes, such as influencing gray matter abnormalities.

In out study, Pearson correlations were used as an initial exploratory analysis to assess the strength and direction of simple pairwise associations between mean FA and cognitive test results. GLM regressions were then employed to examine these relationships while simultaneously controlling for potential confounding factors and testing the unique contribution of each predictor within a multivariate framework. The results from the two approaches were not always concordant. For example, Conceptual level response was associated with mean FA under Pearson correlation analysis, but the association was ruled out with GLM regression.

In the regression models predicting cognitive test performance, we obtained no significant results when group as a discreet approach was replaced with illness duration as a continuous approach. Our explanation was the continuous approach had no large enough power to differentiate the participants on a one-year division of illness duration basis. An illness duration approach requires many more participants than our study [68].

### Two steps of white matter change

Since cognitive impairment occurred before reduction in integrity of individual white matter tracts in our study, a reasonable explanation was, according to the disconnection hypothesis, [69] white matter integrity reduction may be predated by alteration in network organization. By using graph theoretical analysis, we found several global network indices changed in the individuals with schizophrenia with illness duration less than 5 years, which was much earlier than reduction in white matter integrity. In line with several previous studies, including a meta-analysis, we found decreased clustering coefficient and decreased local efficiency, which means reduction in segregation, and increased characteristic path length and decreased global efficiency, which means reduction in integration, in the participants with schizophrenia [32, 33, 70]. Reductions in local segregation and global integration lead to a higher-cost yet lower-efficient network in schizophrenia [71]. However, the global network indices did not continue to change at later stages of illness in our study, which was discordant with the further cognitive impairment. Therefore, neither white matter integrity reduction or network organization deficit alone was fully concordant with cognitive deterioration in schizophrenia. Differential longitudinal changes in white matter integrity and network organization in schizophrenia were also reported by a previous study [72]. In light of these findings, we thought white matter organization abnormalities associated with cognitive deterioration in schizophrenia may have two steps of change. First, network organization alters at early stage of illness despite of preserved integrity of white matter tracts (disorganization of wiring), [73] and second, white matter integrity reductions ensue and spread to almost all white matter tracts at later stages (corrosion of wires). As to white matter integrity reductions, initially in the second decade of the illness course, corpus callosum, corona radiata, and internal capsule are impaired; in the third decade, the impairment has occurred in almost all white matter tracts, which is in line with previous research [69]. Some research reported FA reductions occur at earlier stages of schizophrenia than ours. This difference may be due to relatively stabilized symptomatology of our participants (manifested with lower PANSS scores). Our study is the first one to show deficits in white matter network organization (measured with graph indices) occur earlier in illness course of schizophrenia than reductions in white matter tracts integrity (measured with fractional anisotropy).

### Limitations

This study has several limitations. First, since this is a cross-sectional study, longitudinal changes in cognitive function and white matter organization could only be indirectly inferred. However, since a longitudinal study of patients throughout a three-decade course of schizophrenia is not feasible, a cross-sectional one using groups of participants with different illness durations is a reasonable alternative. Second, like other studies on medicated patients with schizophrenia, we could hardly rule out antipsychotics effect on cognition and the brain since it has been extensively documented [74]. However, by using dose-years to measure cumulative exposure, we found no significant correlation between antipsychotics use and cognitive function or white matter abnormalities. Third, this study focused on white matter as correlate of cognitive function without considering the role of gray matter, both of which may be a part of a greater picture of brain abnormalities in schizophrenia. Fourth, this study used only structural imaging and could not detect brain abnormalities revealed by functional imaging.

## Conclusion

We used a cross-sectional approach of recruiting participants with different illness duration to simulate a longitudinal study tracing changes in cognitive function and white matter organization across the illness course of schizophrenia. We found cognitive impairment and associated white matter organization abnormalities emerged at early stage of schizophrenia and aggravated until the third decade of illness. White matter organization abnormalities manifested initially with deficits in network organization of white matter and then with reductions in white matter tract integrity. Our findings supported the traditional concept of dementia praecox and the promising disconnection hypothesis of schizophrenia. This study also suggests active cognitive rehabilitation to patients with schizophrenia throughout the course of illness may be warranted.

## Supplementary Information

Below is the link to the electronic supplementary material.


Supplementary Material 1

