# Supplementary material for: Cognitive impairment and associated white matter organization abnormalities across the illness course of schizophrenia
Source: Eur Arch Psychiatry Clin Neurosci. 2025 Dec 8;276(6):2839–50. doi: 10.1007/s00406-025-02161-2 (PMC13525951; doi:10.1007/s00406-025-02161-2)
Supplement: Supplementary file 1 [file 406_2025_2161_MOESM1_ESM.docx]

**Supplement Table 1** White Matter Tracts of 5-year SZ, 15-year SZ and 25-year SZ with Different FA from HC

| **5-year SZ** | **15-year SZ** | **25-year SZ** |
| --- | --- | --- |
|  | Genu of corpus callosum | Mid. cerebellar peduncle |
|  | Body of corpus callosum | Pontine crossing tract |
|  | Splenium of corpus callosum | Genu of corpus callosum |
|  | Ant. corona radiata L | Body of corpus callosum |
|  | Sup. corona radiata L | Splenium of corpus callosum |
|  | Cingulum cingulate gyrus R | Fornix column and body of fornix |
|  | Post. limb of int. capsule L | Medial lemniscus L |
|  |  | Inf. cerebellar peduncle R |
|  |  | Inf. cerebellar peduncle L |
|  |  | Sup. cerebellar peduncle R |
|  |  | Sup. cerebellar peduncle L |
|  |  | Cerebral peduncle R |
|  |  | Cerebral peduncle L |
|  |  | Ant. limb of int. capsule R |
|  |  | Ant. Limb of Int. Capsule L |
|  |  | Post. limb of int. capsule R |
|  |  | Post. limb of int. capsule L |
|  |  | Retrolenticular R |
|  |  | Retrolenticular L |
|  |  | Ant. corona radiata R |
|  |  | Ant. corona radiata L |
|  |  | Sup. corona radiata R |
|  |  | Sup. corona radiata L |
|  |  | Pos. corona radiata L |
|  |  | Post. thalamic radiation R |
|  |  | Post. thalamic radiation L |
|  |  | Sagittal stratum R |
|  |  | Sagittal stratum L |
|  |  | External capsule R |
|  |  | External capsule L |
|  |  | Cingulum cingulate gyrus R |
|  |  | Cingulum cingulate gyrus L |
|  |  | Cingulum hippocampus R |
|  |  | Cingulum hippocampus L |
|  |  | Fornix cres stria terminalis R |
|  |  | Fornix cres stria terminalis L |
|  |  | Sup. longitudinal fasciculus R |
|  |  | Sup. longitudinal fasciculus L |
|  |  | Sup. fronto occipital fasciculus R |
|  |  | Sup. fronto occipital fasciculus L |
|  |  | Uncinate fasciculus R |
|  |  | Uncinate fasciculus L |
|  |  | Tapetum R |
|  |  | Tapetum L |

SZ, participants with schizophrenia; HC, healthy controls.

**Supplement Table 2** White Matter Tracts with Significant Pearson’s Correlation of FA with WCST

|  | **HC** | **SZ** |
| --- | --- | --- |
| Trials administered | Left Tapetum | Left Uncinate fasciculus |
| Total correct | Global white matter tracts (mean FA)  Fornix column and body of fornix  Right Superior cerebellar peduncle | Global white matter tracts (mean FA)  Middle cerebellar peduncle  Pontine crossing tract  Right Corticospinal tract  Left Corticospinal tract  Left Medial lemniscus  Right Inferior cerebellar peduncle  Left Inferior cerebellar peduncle |
| Total errors |  | Middle cerebellar peduncle  Right Corticospinal tract  Left Corticospinal tract  Left Medial lemniscus |
| Perseverative responses | Global white matter tracts (mean FA)  Fornix column and body of fornix | Middle cerebellar peduncle |
| Perseverative errors | Fornix column and body of fornix |  |
| Non-perseverative errors | Left Superior cerebellar peduncle |  |
| Conceptual level responses | Global white matter tracts (mean FA)  Fornix column and body of fornix | Global white matter tracts (mean FA)  Right Corticospinal tract  Left Corticospinal tract  Left Medial lemniscus  Right Inferior cerebellar peduncle  Left Inferior cerebellar peduncle |
| Categories completed |  | Middle cerebellar peduncle  Pontine crossing tract  Right Corticospinal tract  Right Anterior Coronal radiata  Right Frontal  occipital fasciculus  Right Tapetum  Left Tapetum |
| Trials to completed 1st category | Right Anterior Coronal radiata | Global white matter tracts (mean FA)  Middle cerebellar peduncle  Pontine crossing tract  Right Corticospinal tract  Left Corticospinal tract  Left Medial lemniscus  Right Inferior cerebellar peduncle  Left Inferior cerebellar peduncle |
| DSF | Splenium of corpus callosum  Right Anterior limb of internal capsule  Left Anterior limb of internal capsule  Right Retrolenticular  Left Retrolenticular |  |
| DSB |  | Middle cerebellar peduncle  Left Corticospinal tract  Left Medial lemniscus |
| MMSE |  | Middle cerebellar peduncle  Pontine crossing tract  Right Corticospinal tract  Left Corticospinal tract  Left Medial lemniscus  Left Inferior cerebellar  peduncle |

WCST, Wisconsin Card Sorting Test; DSF, Forward Digit Span; DSB, Backward Digit Span. SZ, participants with schizophrenia; HC, healthy controls.

**Supplement Figure 1** Summary of General Linear Models Examining the Association of Cognitive Test Results with mean FA

**SZ (using Group as a predictor)**

SZ (using Illness duration as a predictor)

HC

SZ, participants with schizophrenia; HC, healthy controls.

p < 0.05.
